# Tissue Dependent Role of PTX3 During Ischemia-Reperfusion Injury

**DOI:** 10.3389/fimmu.2019.01461

**Published:** 2019-07-10

**Authors:** Thiago Henrique Caldeira de Oliveira, Danielle G. Souza, Mauro Martins Teixeira, Flávio Almeida Amaral

**Affiliations:** ^1^Immunopharmacology Laboratory, Department of Biochemistry and Immunology, Universidade Federal de Minas Gerais, Belo Horizonte, Brazil; ^2^Host-Microorganism Interaction Laboratory, Department of Microbiology, Universidade Federal de Minas Gerais, Belo Horizonte, Brazil

**Keywords:** PTX3, ischemia and reperfusion injury, sterile inflammation, hypoxia, adhesion moleculaes, neutrophil

## Abstract

Reperfusion of an ischemic tissue is the treatment of choice for several diseases, including myocardial infarction and stroke. However, reperfusion of an ischemic tissue causes injury, known as Ischemia and Reperfusion Injury (IRI), that limits the benefit of blood flow restoration. IRI also occurs during solid organ transplantation. During IRI, there is activation of the innate immune system, especially neutrophils, which contributes to the degree of injury. It has been shown that PTX3 can regulate multiple aspects of innate immunity and tissue inflammation during sterile injury, as observed during IRI. In humans, levels of PTX3 increase in blood and elevated levels associate with extent of IRI. In mice, there is also enhanced expression of PTX3 in tissues and plasma after IRI. In general, absence of PTX3, as seen in PTX3-deficient mice, results in worse outcome after IRI. On the contrary, increased expression of PTX3, as seen in PTX3 transgenic mice and after PTX3 administration, is associated with better outcome after IRI. The exception is the gut where PTX3 seems to have a clear deleterious role. Here, we discuss mechanisms by which PTX3 contributes to IRI and the potential of taming this system for the treatment of injuries associated with reperfusion of solid organs.

## Introduction

Impaired blood flow to tissues caused by reduced or obstructed arterial inflow (ischemia) and consequent decreasing of oxygen and nutrient supply is an intrinsic condition during clinical procedures, including coronary angioplasty, vascular reconstruction, organ transplantation, and vascular diseases, such as stroke, myocardial, renal, and intestinal infarction (1–3). Although reperfusion brings blood flow and oxygen back, which are essential to prevent irreversible tissue injury, it may paradoxically worsen ischemic tissue damage. During reperfusion, there is excessive production of pro-inflammatory molecules by the ischemic tissue and systemic distribution of these molecules. This phenomenon is known as ischemia-reperfusion injury (IRI) and is a major issue during organ transplantation, as it directly correlates to graft rejection (4–6). IRI is responsible for up to 10% of early transplant failures and is also associated with high rates of acute and chronic rejection (7–9).

During ischemia, adenosine triphosphate (ATP) production is impaired due to decreased oxygen supply (10). In addition, ischemic tissue produces high levels of pro-inflammatory cytokines, vasoactive agents, adhesion molecules, and reactive oxygen species (ROS) (4). Particularly, ROS generation modifies intracellular pH that is associated with organelle damage and cell death (11). In this sterile inflammatory context, the innate immune response is activated when dead cells release their contents into the extracellular environment, which are recognized by pattern recognition receptors (PRRs) expressed on resident immune cells. Furthermore, soluble pattern recognition molecules work as fluid-phases receptors, distributed mainly in distinct liquid compartments. This humoral arm of the innate immune system consists of three clearly defined subgroups of molecules, represented by collectins, ficolins, and pentraxins (12).

Pentraxins belong to a family of phylogenetically conserved proteins and are divided into two groups according to the length of their primary structure: the short and long chain pentraxins (13). The classical short pentraxins, represented by C-reactive protein (CRP) and serum amyloid P component (SAP) are produced in the liver under pro-inflammatory stimuli, most prominently by IL-6. Both CRP and SAP bind to different ligands of microbes and host components in a calcium-dependent manner, a mechanism associated to innate immunity against pathogens and also for scavenging of cellular debris (14). Long pentraxins are characterized by an unrelated N-terminal domain coupled to a pentraxin-like C-terminal domain (15). The prototypic long pentraxin 3 (PTX3), also formerly referred to as TSG-14 (TNF-stimulated gene 14) was identified in the early 1990s in human endothelial cells and fibroblasts as a TNF or IL-1β-inducible mRNA and protein, respectively (16, 17). Here, we provide an overview of currently available data about the role of PTX3 in the complex mechanisms involved in the immune response during IRI in different organs. Then, we discuss possible options for IRI therapy based on the knowledge of PTX3 biology.

## Mechanisms of Tissue Damage During Ischemia and Reperfusion Injury (IRI)

Several pathological processes contribute to IRI, including impaired endothelial cell barrier function (18, 19), activation of cell death programs (20) and activation of innate and adaptive immune responses (21). IRI occurs as the result of a biphasic condition. During ischemia, when the oxygen levels decrease, there is a dysfunction of the electron transport chain in mitochondria and a shift from aerobic to anaerobic metabolism, which impairs ATP production. Moreover, there is accumulation of lactic acid and ketone bodies, leading to decrease of pH in tissues and cells, known as metabolic acidosis. The lack of energetic substrate also interferes with transmembrane transports, causing dysfunction of sodium-potassium and calcium pumps on the cell surface, which results in cell hyperosmolarity and flow of water into the cytoplasm and cell swelling (22). In ischemic tissues, a large number of ROS are produced by mitochondria. ROS production can cause damage to membrane lipids, proteins, and DNA, leading to endothelial cell dysfunction and consequently cell death (23). In addition, the deleterious effects of low oxygen levels spread along different cell types in the affected tissue (24–27). There is a variation of the resistance to ischemia among cell populations of a given tissue. For example, cardiac cells are more resistant to periods of ischemia as compared to hepatocytes and Kupffer cells (28).

The magnitude and duration of ischemia will determine the degree of cell dysfunction and death. Cells that died during the ischemic phase release a range of intracellular molecules called danger associated molecular patterns (DAMPs), also known as alarmins. Under homeostatic conditions, these molecules are hidden into intracellular compartments. However, under conditions of cellular stress, DAMPs are released to the extracellular environment or kept on cell membrane (29). Different molecules have been described as DAMPs, including ROS, ATP, high mobility group box 1 (HMGB1), DNA, mitochondrial formyl peptides, IL-1, urate, and S100 proteins. These molecules bind to a variety of PRR and trigger inflammatory responses through the activation of various signaling pathways (30). Innate immune, parenchymal and endothelial cells express PRRs on their surface and in their cytoplasm, which recognize DAMPs. PRRs include Toll-like receptors (TLRs), Retinoic Acid-Inducible Gene I-like receptors, nucleotide-binding oligomerization domain-like receptors (NLRs), including the inflammasomes, and C-type lectin receptors. Thus, it seems clear that DAMPs released during ischemia contribute to the intense inflammatory response seen in IRI (31).

The reperfusion phase occurs when the blood flow is restored to the ischemic tissue. During the first minutes, the blood flow to ischemic tissue may not happen immediately, a phenomenon known as no-reflow. It is believed that this intravascular obstruction may be caused by leukocytes and platelets (32). Although reperfusion is required to restore oxygen to the tissue, the metabolic distress caused during ischemia creates a condition that triggers a set of excessive innate immune response, which exacerbates the injury to vascular and parenchymal cells during reperfusion. The reperfusion can be separated in two phases. Initially, there is intense oxidant stress, leading to impaired production of antioxidative molecules that increases ROS generation further. ROS initiate a series of cellular events that cause inflammation, promoting cellular injury through endothelial dysfunction, DNA damage, necrosis and/or apoptosis (11). An important pathway of ROS production involves reduction of nicotinamide adenine dinucleotide phosphate (NADPH) oxidase (32). Indeed, abrogation of NADPH component in mice reduces the deleterious effects of IRI (33). In addition, the reperfusion phase sets deposition of complement, upregulation of adhesion molecules, inflammatory cell infiltration, mainly neutrophils, and further pro-inflammatory mediators production (33, 34). The local presence of DAMPS and molecules produced in response to DAMPs will feed into this increasing inflammatory reaction seen during IRI.

Although the degree of injury may vary in different tissues, a common feature in all organs is microvascular dysfunction. The vascular injury induced by IR is a consequence of local and systemic inflammatory response and includes vascular permeability, endothelial cell activation, platelet–leukocyte interaction, complement activation, and imbalance between vasodilating and vasoconstricting factors (3). Tissue hypoxia during ischemia directly influences the increase of vascular permeability, as demonstrated by studies with endothelial cells exposed to an environment with low oxygen concentration, a phenomenon that alters endothelial cell barrier function in a mechanism dependent on reduced adenylate cyclase activity and intracellular cAMP levels (19). Moreover, studies *in vivo* demonstrated that animals exposed to a hypoxic environment showed vascular leakage in multiple organs and increased hypoxia-associated pulmonary edema (35, 36).

The migration of neutrophils from blood into tissue during vascular inflammation occurs by a multistep cascade. There is initial tethering and rolling on vessel wall via selectins interactions followed by firm adhesion and emigration out of the vasculature to the parenchyma. These events are well-established in different microvasculatures including the peritoneum, mesentery, skeletal muscle, and skin (37). In this regard, tissue and resident cells produce chemoattractant factors, such as chemokines, that guide neutrophil infiltration into the site of inflammation. To induce neutrophil migration, chemokines are maintained in high concentration on the endothelium cell surface by binding to glycosaminoglycans (38). Moreover, other factors contribute to neutrophil migration and microvascular dysfunction after reperfusion, including complement components and leukocyte interactions with platelets (39). Upon leaving the vessels and entering the tissues, activated leukocytes release ROS and proteases, causing increased microvascular permeability, edema, thrombosis, and parenchymal cell death (39). Indeed, previous reports have shown that Reparixin, a non-competitive allosteric antagonist of chemokine receptor CXCR2 was able to prevent neutrophil migration and reduce liver and intestinal damage, suggesting that excessive neutrophil migration is detrimental to tissues following reperfusion (40, 41).

## Role of PTX3 During Sterile Inflammation

**Figure 1:**
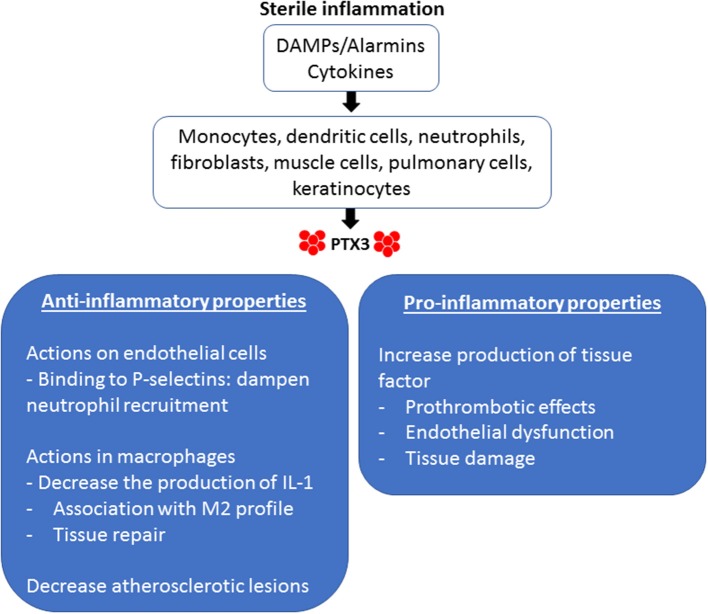
Pro-inflammatory and anti-inflammatory properties of PTX3 in sterile inflammation. Endogenous stimuli induce the production of PTX3 by different cellular types. DAMPs, Damage-associated molecular patterns.

There are several actions of PTX3 that are relevant to the sterile inflammation that occurs during IRI (Figure 1). PTX3 can be considered an acute-phase protein. In normal conditions, its serum levels is low (around 25 ng/ml in the mouse, <2 ng/ml in humans), but quickly increases during inflammation (200–800 ng/ml in humans and mice) (42). Innate immune factors stimulate the production of PTX3 locally, including pattern molecules (DAMPs) and cytokines (43). Particularly in the field of sterile inflammation, IL-1 is a potent inducer of PTX3 production during tissue damage, as occurs in mouse models of acute myocardial infarction (AMI) (44).

In terms of kinetics, local production of PTX3 begins before the synthesis of classical pentraxins by hepatic parenchyma cells. PTX3 can be produced in several organs, especially in the heart and striated muscle, but also in the lungs, ovarium, thymus, and the skin (45). Resident leukocytes and parenchymal cells are important sources of PTX3 (46, 47). PTX3 is also stored in neutrophil granules, which are released under inflammatory stimuli. It is estimated that neutrophils release about 25% of their PTX3 to the extracellular compartment, much of them associated with neutrophil extracellular traps (NETs) (48). Thus, while CRP and SAP are produced in the liver and carried to the inflammatory foci by blood flow, PTX3 is formed locally at sites of ongoing inflammatory reaction (45, 49).

The multifunctional properties of PTX3 include interactions with different ligands, such as complement C1q component, the extracellular matrix component TSG6, apoptotic cells, endothelial cells, and leukocytes (50). Complement factors and PTX3 have been considered important regulators in the clearance of dying cells. In this regard, an investigation in mammalian cells showed that soluble PTX3 binds to immobilized C1q and, reciprocally, C1q bound to immobilized PTX3 (51). In addition, C1q and PTX3 present different functions during the phagocytosis of apoptotic cells. Previous reports have demonstrated that complement C1q is necessary for effective phagocytosis of apoptotic cells by macrophages, whereas PTX3 inhibits this process (52, 53). This mechanism was elucidated by Baruah and coworkers' study, who showed that C1q and PTX3 have different affinity for apoptotic cell domains. However, the presence of PTX3 in the solution removed bound C1q from apoptotic cells, leading to inhibition of complement activation by C1q on apoptotic cells and their phagocytosis by dendritic cells. Moreover, it has been shown that although PTX3 decreased the internalization of dying cells by human dendritic cells, it did not affect the capture of soluble or inert particulate substrates, such as fluorescent ovalbumin and latex beads. Furthermore, apoptotic cells preincubated first with PTX3 did not modify binding of C1q to these dying cells. Thus, these findings suggest that PTX3 and C1q interaction may occur in soluble phase, reducing the availability of C1q-mediated phagocytosis (54). These results suggest that although PTX3 prevents cell phagocytosis by dendritic cells, it favors the sequestration of cell debris by antigen-presenting cells, which could contribute to reduce self-antigen presentation and a possible development of autoimmune disorders (55). Furthermore, deficiencies of C1q is associated with development of systemic erythematosus lupus (SLE) and accumulation of apoptotic cells in renal glomeruli, which emphasizes the importance of C1q in the clearance of cellular debris (56). It has been demonstrated for a long time that patients with SLE have a well-characterized defect in the production of pentraxins during active phases of the disease (57). Thus, these data suggest that the interaction of C1q and PTX3 may have important implications in the healthy removal of cellular debris under inflammatory conditions and protection against autoimmunity.

PTX3 also interacts with endothelial cell adhesion molecules. Deban and coworkers reported that PTX3 released by hematopoietic cells prevent excessive neutrophil recruitment under P-selectin interaction. This observation was also demonstrated using exogenous PTX3.This finding suggests a natural anti-inflammatory effect of PTX3 in P-selectin-dependent models of leukocyte recruitment and inflammation (58). Moreover, models of sterile inflammation, such as AMI induced by coronary artery ligation and reperfusion or cerebral IRI, showed that absence of PTX3 was associated with increased neutrophil migration and tissue damage (44, 59). These results suggest that PTX3 provides a feedback loop by preventing neutrophil recruitment and tissue damage in models of sterile inflammation. In addition, PTX3 influences macrophages function. PTX3 impairs the production IL-1β, TNF, and CCL2 levels, whilst stimulates TGF-β production by THP-1 macrophages. These results were associated with Akt phosphorylation and reduced NF-κB activation in the presence of PTX3. Silencing PTX3 increased IL-1β production by macrophages (60). Moreover, it has been reported that mice lacking PTX3 subjected to wire-mediated endovascular injury exhibited higher deteriorated neointimal hyperplasia after vascular injury via the effects of macrophage accumulation (61). Thus, considering the function of PTX3 in control pro-inflammatory molecules production by macrophages, half of macrophages positive for PTX3 in coronary atherosclerosis presented M2-like phenotype (62). Therefore, all these findings suggest a role for PTX3 in resolving inflammation by suppressing the activity of macrophages at inflamed sites and inducing healing process.

Recent studies using genetic-modified mice demonstrated that PTX3 has an important action in regulating vascular sterile inflammation. Norata and coworkers have shown increased expression of PTX3 in the vasculature during atherogenesis. Mice deficient for PTX3 fed with an atherogenic diet showed larger atherosclerotic lesions compared with WT mice. These mice also showed increased expression of adhesion molecules, cytokines, and chemokines in the vascular wall, associated with intense accumulation of macrophages within atherosclerotic plaque (63). On the other hand, although these results suggest atheroprotective and cardiovascular protective effects of PTX3 by modulating the vascular-associated inflammatory response, this molecule induces tissue factor in endothelial cells, presenting potential proinflammatory and prothrombotic properties (64, 65). Thus, PTX3 may orchestrate different roles depending on the scenario of vascular pathology. In this regard, increased levels of PTX3 is observed in vascular disorders, such as myocardial infarction and small vessel vasculitis that correlate with worsen outcome or disease activity. In fact, during inflammation, blood vessels produce large amounts of PTX3 (66). PTX3 has been linked to vascular endothelial dysfunction in several diseases, including chronic kidney disease and preeclampsia, a condition associated with hypertension (67, 68). Carrizzo and coworkers have shown that PTX3 promotes endothelial dysfunction and morphological changes by a mechanism dependent on P-selectin and matrix metalloproteinase-1 (MMP1) pathway. *In vivo* administration of PTX3 induced endothelial dysfunction and increased blood pressure. Moreover, inhibition of MMP1 protected mesenteric arteries against the endothelial dysfunction promoted by PTX3, an effect absent in P-selectin-deficient mice (69). In addition, overexpression of PTX3 attenuates the production of nitric oxide by a mechanism dependent on the upregulation of MMP1 and P-selectin (69). Therefore, these studies suggest that a high plasma concentration of PTX3 could be a biomarker of altered endothelial function in different diseases.

## Role of PTX3 in Organ Specific IRI

**Figure 2:**
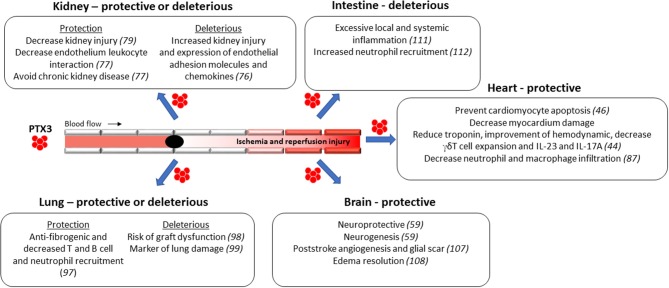
Protective and deleterious functions of PTX3 during Ischemia and Reperfusion Injury (IRI) in different organs. The role of PTX3 during IRI seems to be organ specific, depends on the amount and source of this protein, and the related disease.

In the last decades, the mechanisms associated to IRI pathogenesis has been extensively investigated, although they have not yet been completely elucidated. As discussed above, IRI is characterized by intense tissue inflammation due to high production of local pro-inflammatory cytokines and with massive accumulation of neutrophils. As described below, the role of PTX3 during IRI seems to be organ specific, depends on the amount and source of this protein, and the related disease (70–73) (Figure 2).

### Renal IRI

Renal IRI syndrome develops after a sudden transient decrease in total or regional blood flow to the kidney (74). The sterile inflammatory disease observed in this condition occurs due to endothelial cell activation caused by endothelial cell-leucocyte interaction and by reduced vascular blood flow. In addition to endothelial cell damage, IRI is associated with endothelial-leukocyte interactions through the up-regulation of adhesion molecules.

A previous study has shown that injured renal cells released endogenous HMGB1 after IRI. HMGB1 binds to endothelial Toll-like receptor 4 (TLR4), promoting an increase of adhesion molecules expression in vasculature (75). Interestingly, PTX3 is regulated by TLR4 activation, since TLR4-deficient mice subject to renal IRI showed reduced PTX3 production and lower renal damage when compared with WT animals. This modification in PTX3 expression occurred together with other markers of endothelial activation and was associated to reduced kidney injury and lower expression of endothelial adhesion molecules and chemokines when compared to wild type mice (76).

Although the study above suggested a deleterious role of PTX3 in the context of IRI, others provide more direct evidence for a beneficial role of this protein. Renal injury was aggravated in PTX3-deficient mice subject to IRI by a mechanism dependent on the control of neutrophil and macrophage recruitment into the postischemic kidney (77). Mechanistically, absence of PTX3 could affect PTX3-P-selectin interaction (58). The neutralization of P-selectin by specific antibody completely abrogated IRI-induced tissue damage. Interestingly, administration of recombinant PTX3 injection in the reperfusion phase effectively prevented renal inflammation, as observed by reduction of leukocyte accumulation by suppressing leukocyte-selectin interaction and consequent leukocyte rolling on endothelial cells (77). In this regard, lack of PTX3 increase the expression of P-selectin, favoring the interaction of circulating leukocytes with activated endothelial cells (78). Thus, in the kidney undergoing IRI injury, local PTX3 production tends to avoid excessive organ inflammation and dysfunction. In addition, PTX3 injection recovered kidney function as observed by the reduction of IRI-induced interstitial fibrosis by a mechanism associated with the reduction of IL-6 and p-STAT3 (79).

### Cardiac IRI

Cardiovascular diseases (CVD) are responsible for high number of deaths in the developed world and numerous studies have indicated that PTX3 has a potential contribution to prevent the progression of CVD (80, 81). The reperfusion of affected coronary arteries is a crucial step for an effective therapy after a myocardial infarction. However, as it occurs in other organs, the restoration of blood flow is associated to myocardium damage that limits the benefit of blood flow restoration, known as myocardial IRI. A similar phenomenon is also seen during cardiac transplantation, which is associated with organ dysfunction that impairs cardiac recovery (82). Local inflammation is the major problem that contributes to cardiac IRI, associated with intense and rapid production of cytokines and accumulation of leukocytes in the affected area. Neutrophils migrate rapidly to the infarct zone guided by chemoattractants during the first 24 h of myocardial IR and release degradative enzymes that contribute to irreversible myocardial damage (83). PTX3 can be released by neutrophils early and by macrophages and endothelial cells in the late phase of myocardial infarcted patients (84) and there is evidence to suggest that the heart is a major site for PTX3 expression (85), which could contribute to its involvement in multiple cardiovascular disorders.

A transcriptomic analysis of the whole blood obtained after cardiac surgery identified PTX3 as a potential indicator for infarction and irreversible injury of the myocyte in ischemic cardiomyopathy (86). Using an experimental model of myocardial infarction and samples of myocardial infarction of patients, Maugeri and coworkers demonstrated that neutrophils were the main source of increased PTX3 in blood of patients with AMI in the early phase of the symptoms (within 6 h). Moreover, activated platelets were responsible to trigger neutrophil PTX3 release. Indeed, a substantial fraction of PTX3 was observed on cell membranes of circulating platelets in patients with AMI. In the presence of PTX3, the formation of platelet-neutrophil aggregation was inhibited, which was associated to less effectiveness of platelets at upregulating CD11b/CD18 integrin expression, a critical step for leukocytes to adhere to and transmigrate within inflamed tissues (87). These results suggested that PTX3 decreases the inflammatory response triggered by activated platelets, limiting noxious effects of neutrophils in the heart.

Other studies have also suggested that PTX3 has important functions for the protection of AMI. For instance, exogenous PTX3 played a protective role in myocardial IRI by preventing cardiomyocyte apoptosis and reducing troponin production in mice, which was associated to an improvement of hemodynamic performance (46). That study also demonstrated an important effect of PTX3 on cell function, restricting γδT cell expansion and activation, decreasing local expression of the proinflammatory cytokines IL-23 and IL-17A and neutrophil and macrophage infiltration in the tissue. Furthermore, using an experimental model of acute cardiac ischemia and reperfusion in mice, researchers identified a kinetics of PTX3 mRNA in the circulation which peaked after 24 h and returned to basal levels after 3 days (44). In the same study, mice deficient for PTX3 presented increased myocardial damage after cardiac IRI, with extended area without reflow, intense accumulation of leukocytes into affected area, and elevated number of apoptotic cardiomyocytes. Interestingly, the infusion of exogenous PTX3 in these mice reversed that phenotype (44). Thus, PTX3 seems to have a protective role to reduce myocardium damage by reducing heart inflammation. In addition to its potential therapeutic role, it is suggested that PTX3 could be used as an early indicator of CVD and an important inflammatory component of ischemic heart disease in humans. Peri and coworkers demonstrated that plasma levels of PTX3 were elevated after myocardial infarction faster than C-reactive protein, suggesting that PTX3 could be used as an earlier indication of cardiac IRI (88). PTX3 is present in normal cardiomyocytes (88). The increased PTX3 in blood may be a consequence of its release from dying or necrotic cells due to increased permeability of necrotic cardiomyocyte (89).

### Pulmonary IRI

Pulmonary IRI frequently occurs during lung transplantation, especially in the earlier stages of transplantation, as a form of acute lung injury (ALI) (90). Importantly, the development of ALI in the first 3 days after lung transplantation is associated to the development of chronic lung allograft dysfunction (CLAD), a condition that reduces up to 50% survival in the first 5 years after surgery (91–93). In ALI and acute respiratory distress syndrome patients, plasma PTX3 is elevated and is positively correlated with lung injury parameters (94). In transplantation models, IRI has been directly related to the activation of the innate immune system, which involves recognition TLR signaling pathways, complement activation and natural killer cell migration in transplantation models, and leads to decreased allograft tolerance in many organs (95, 96). In the lung, IRI leads to five main processes that result in regional injury, including sterile immunity, activation of coagulation, activation of cell death pathways and endothelial dysfunction (97).

A few studies indicate that PTX3 has protective effects in lung IRI. For instance, PTX3-deficient mice subjected to orthotopic lung transplantation showed increased lung parenchymal fibrosis 28 days after lung transplantation. These mice had significantly larger numbers of T cells and B cells, which is associated with CLAD (98). This is in line with others models of ALI, where PTX3 dampened neutrophil extravasation to lung parenchyma, while PTX3-deficient mice had worsen lung injury (58). Thus, these results indicate acute beneficial effects of PTX3 in lung transplant recipients and protection against the development of chronic rejection. On the other hand, a study by Diamond and colleagues reported that patients with idiopathic pulmonary fibrosis and chronic obstructive pulmonary disease showed higher levels of PTX3 6 h and 24 h after reperfusion when compared with controls. Moreover, there was a positive correlation between elevated PTX3 levels and the elevated risk of graft dysfunction in lung transplant recipients with idiopathic pulmonary fibrosis (99). In this sense, PTX3 could be used as a marker of lung damage and severity of disease since is quickly detected in ALI patients (94). It is important to mention the differences among those studies. The protective role of PTX3 was performed mice. In humans, there was only a positive correlation between PTX3 levels and lung injury. Indeed, there are no data to explain whether increased levels of PTX3 are protective or harmful, as demonstrated above in cardiac IRI, where elevated PTX3 levels promote negative feedback on the inflammatory response to the heart (46). Thus, it is not possible to define a causal relationship between PTX3 release leading to lung injury in humans.

### Brain IRI

Different organs exhibit different levels of susceptibility to IRI with the brain being perhaps the most IRI sensitive organ, as irreversible brain damage can become evident within 20 min of ischemia (100). Cerebral ischemia is associated with high mortality and disability rates worldwide, as evidenced in stroke, intracerebral or subarachnoid hemorrhage, traumatic brain injury or perinatal hypoxia, and the intense production of pro-inflammatory mediators in acute cerebral ischemia is directly associated with brain damage (101). Increased number of circulating leukocytes and intense recruitment of neutrophils to the brain can be observed up to 24 h after the first symptoms of stroke (102). In addition, the inflammatory state at the affected site is associated to high levels of cytokines, including IL-1β, IL-6, TNFα, IL-10, TGF-β, and chemokines, such as CCL2, CCL3, CXCL1, and CX3CL1 (101, 103). Among them, IL-1β has been considered critical for the brain inflammation after stroke. Its expression is rapidly produced and contributes to brain neurotoxicity. In addition, the blockade of IL-1 receptor prevents ischemic and excitotoxic neuronal damage in rat (104).

As observed in other tissues, PTX3 has been considered a new mediator of inflammation in cerebrovascular disorders and also be considered a potential prognostic marker in ischemic stroke (105). Early after ischemic stroke, peri-infarct astrocytes are important source of PTX3 (106, 107). The production of PTX3 in brain is dependent on IL-1β release after cerebral ischemia and it mediates the formation of the glial scar and resolution of brain edema. Interestingly, mice deficient for PTX3 had marked increase in tissue damage and unresolved cerebral edema after 6 days of cerebral ischemia (108). In accordance, PTX3 deletion impaired blood brain barrier integrity, increased brain inflammation and decreased the resolution of tissue damage (108). Another report showed that PTX3-deficient mice subjected to experimental cerebral ischemia showed reduced neurogenesis in the dentate gyrus of the hippocampus. Furthermore, absence of PTX3 was associated to marked reduction in poststroke angiogenesis when compared to wild type mice 2 weeks after cerebral ischemia. In addition, recombinant PTX3 demonstrated important neurogenic role *in vitro* (59). These data indicate that PTX3 contributes to recovery after stroke through regulation of neurogenesis and angiogenesis and glial scar formation.

### Intestinal IRI

Intestinal ischemia occurs following mesenteric artery blockade with consequent reduction of blood flow to the area. Gust ischemia is very lethal and reperfusion is the only therapy of choice in these cases and may culminate in intense intestinal tissue inflammation and damage. Different conditions and procedures may cause intestinal ischemia, including necrotizing enterocolitis, allograft rejection in small bowel transplantation, complications of abdominal aortic aneurysm surgery, cardiopulmonary bypass, and inflammatory bowel disease (3, 109). Another critical point during intestinal IRI is the risk of loss of the intestinal barrier, facilitating bacterial translocation into the circulation, that could be associated with the development of sepsis (110).

To date, two studies have addressed the role of PTX3 in the context of intestinal IRI. The first one showed that transgenic mice overexpressing up to 4 extra copies of PTX3 had reduced survival rate after intestinal IRI when compared to wild type mice. This phenotype was associated with increased production of proinflammatory cytokines locally, systemically, and in the lungs (remote organ). This was accompanied by intense tissue damage and hemorrhage in both intestine and remote tissue, as observed in lungs (111). In addition, PTX3-deficient mice were protected from intestinal IRI. In PTX3-deficient mice, there was decreased NF-kB translocation and TNF and CXCL1 production when compared to wild type mice. The reduced inflammation was associated with decreased neutrophil influx, preservation of intestinal architecture and significant prevention of lethality. To assert the deleterious effect of PTX3 during intestinal IRI, intravenously infusion of PTX3 reversed the protected phenotype in PTX3-deficient mice (112). Thus, those results show that endogenous PTX3 is essential for the cascade of events leading to tissue inflammation and injury after IR. Moreover, they suggest that PTX3 blockade may be useful as therapy for intestinal IRI.

## Concluding Remarks

PTX3 has clear role in the induction of sterile inflammation, as observed during IRI (Figure 2). In humans, levels of PTX3 increase in blood and elevated levels associate with extent of IRI. In general, absence of PTX3, as seen in PTX3-deficient mice, results in worse outcome after IRI. On the contrary, increased expression of PTX3, as seen in PTX3 transgenic mice and after PTX3 administration, is associated with better outcome after IRI. The overall protective effects of PTX3 are associated with decreased local edema formation and decreased neutrophil-endothelial cell interactions. As neutrophils contribute significantly to IRI, these effects of PTX3 may underlie its beneficial effects in these models. In this regard, it the administration of PTX3 may be beneficial in patients undergoing IRI.

The situation is dramatically different in a model of intestinal IR injury. In the latter model, systemic levels and local expression of PTX3 also increases after reperfusion. However, and in contrast to findings in other systems, decreased PTX3 expression is associated with decreased damage and enhanced expression is associated with more significant and lethal damage in a model of intestinal IR injury (111, 112). It is difficult to reconcile these findings with the overall contrasting effects of PTX3 in models of IR injury in other sites. Intestinal IRI is in general much more severe than IRI to other organs and accompanied by very significant lethality rates within the first few hours after reperfusion. In addition, there is significantly more systemic inflammation and remote damage than in the other models of IRI. There are no studies directly comparing whether local and systemic severity accounts for the differences observed. Regardless of the explanation, it is clear that one should take great caution when considering the administration of PTX3 in instances of severe IRI, as seen in the gut.

## Author Contributions

TdO, DS, MT, and FA designed the article and wrote the manuscript.

### Conflict of Interest Statement

The authors declare that the research was conducted in the absence of any commercial or financial relationships that could be construed as a potential conflict of interest.
